# ISPyB for BioSAXS, the gateway to user autonomy in solution scattering experiments

**DOI:** 10.1107/S1399004714019609

**Published:** 2015-01-01

**Authors:** Alejandro De Maria Antolinos, Petra Pernot, Martha E. Brennich, Jérôme Kieffer, Matthew W. Bowler, Solange Delageniere, Staffan Ohlsson, Stephanie Malbet Monaco, Alun Ashton, Daniel Franke, Dmitri Svergun, Sean McSweeney, Elspeth Gordon, Adam Round

**Affiliations:** a European Synchrotron Radiation Facility, 71 avenue des Martyrs, CS 40220, 38042 Grenoble, France; b European Molecular Biology Laboratory, Grenoble Outstation, 71 avenue des Martyrs, CS 90181, 38042 Grenoble, France; c Unit for Virus Host Cell Interactions, Université Grenoble Alpes–EMBL–CNRS, 71 avenue des Martyrs, CS 90181, 38042 Grenoble, France; d DLS, Diamond House, Harwell Science and Innovation Campus, Fermi Avenue, Didcot OX11 0QX, England; e European Molecular Biology Laboratory, Hamburg Outstation, c/o DESY, Building 25A, Notkestrasse 85, 22603 Hamburg, Germany

**Keywords:** small-angle X-ray scattering, proteins in solution, automation, laboratory information-management system

## Abstract

Logging experiments with the laboratory-information management system ISPyB (Information System for Protein crystallography Beamlines) enhances the automation of small-angle X-ray scattering of biological macromolecules in solution (BioSAXS) experiments. The ISPyB interface provides immediate user-oriented online feedback and enables data cross-checking and downstream analysis. To optimize data quality and completeness, ISPyBB (ISPyB for BioSAXS) makes it simple for users to compare the results from new measurements with previous acquisitions from the same day or earlier experiments in order to maximize the ability to collect all data required in a single synchrotron visit. The graphical user interface (GUI) of ISPyBB has been designed to guide users in the preparation of an experiment. The input of sample information and the ability to outline the experimental aims in advance provides feedback on the number of measurements required, calculation of expected sample volumes and time needed to collect the data: all of this information aids the users to better prepare for their trip to the synchrotron. A prototype version of the ISPyBB database is now available at the European Synchrotron Radiation Facility (ESRF) beamline BM29 and is already greatly appreciated by academic users and industrial clients. It will soon be available at the PETRA III beamline P12 and the Diamond Light Source beamlines I22 and B21.

## Abstract 2

The ISPyB information-management system for crystallography has been adapted to include data from small-angle X-ray scattering of macromolecules in solution experiments.

## 1. Introduction   

### 1.1. A brief history of ISPyB   

In the last 15 years, advances in sample preparation coupled with improvement of synchrotron beamlines and automation of data analysis has gradually led to an increasing need for organizing the deluge of data produced. In 2001, PXWeb, a prototype laboratory information-management system (LIMS) using Zope technology (http://www.zope.org) was developed and deployed at the ESRF (Arzt *et al.*, 2005 ▶). Its function was limited to recording experimental parameters and basic reporting. No data exchange between PXweb and other LIMS was possible. A joint ESRF/e-HTPX (e-science resource for macromolecular crystallography) was then launched (Allen *et al.*, 2003 ▶) and resulted in the development of an upgraded LIMS, ISPyB (Beteva *et al.*, 2006 ▶; Delagenière *et al.*, 2011 ▶). Since 2008, the ESRF has been collaborating with Diamond Light Source (DLS), resulting in a multi-site, generic LIMS for synchrotron-based macromolecular crystallography (MX) experiments. The current version (13.10.2014) allows users to track their sample location during shipping, facilitates the transmission of information to and from other LIMS and records experimental details as well as automatic data-processing results as the experiment proceeds and populates reports of the experimental results (Monaco *et al.*, 2013 ▶). The complexity of experiments increases with the number of samples involved and ISPyB has become a great support for users during their experiment, where it aids rapid decision making as well as maintaining a history of projects over time. Over the years, its role has evolved from an experiment notebook to a central element in macromolecular structure-solution pipelines.

In recent years, BioSAXS has followed the same path as MX in terms of increased automation of experiments with the implementation of sample-changer robots (Round *et al.*, 2008 ▶; David & Pérez, 2009 ▶; Pernot *et al.*, 2010 ▶) and automated online data analysis (Petoukhov *et al.*, 2007 ▶; Hura *et al.*, 2009 ▶; Nielsen *et al.*, 2009 ▶; Kieffer & Karkoulis, 2012 ▶). The modularity of ISPyB allows the data model to easily be extended to include other experimental techniques. BioSAXS is a natural extension, as its requirements for sample tracking and data handling are similar to those for MX and the respective user communities overlap. With the support of BioStruct-X funding in 2012, EMBL Hamburg, DLS and the ESRF set up a collaboration to extend ISPyB to include BioSAXS experiments. The first phase of the project (described in this article) was to develop a generic data model for all SAXS experiments and possible data-processing pipelines. The resulting prototype was implemented on the ESRF beamline BM29 within this collaboration.

### 1.2. Demand for BioSAXS (and required) automation   

BioSAXS experiments are in increasing demand by an ever more diverse research community, both academic and industrial. Even with the increasing level of automation enabling higher throughput, existing facilities were oversubscribed and additional facilities were built to satisfy demand. To better serve the needs of the user community, the dedicated (with rapid access) high-throughput BioSAXS beamline BM29 at the ESRF has been upgraded (Pernot *et al.*, 2013 ▶). This beamline offers fully automated data collection and analysis to facilitate all experimental access modes for structural biology at the ESRF (standard, remote or full service).

Automation of BioSAXS experiments has benefited greatly from the implementation of robotic sample changers (Round *et al.*, 2015 ▶). The ability to handle many samples in an automated way increases throughput and minimizes errors during manipulation. However, preparing a large number of samples can lead to data-entry errors when describing the list of samples in the data-acquisition software. This potentially causes a significant reduction in efficiency and loss of time during an experiment. The need for the storage of sample details and data-acquisition parameters is clear. If experiments are defined in advance and details uploaded on demand to data-acquisition software, the initiation of data collection without any loss of beamtime is possible.

A further extension of ISPyBB is planned with the implementation of automated sample preparation using liquid-handling robotics at high-throughput crystallization (HTX) facilities and parsing sample details from their internal databases such as CRIMS (CRystallization Information Management System; https://embl.fr/htxlab) to ISPyBB.

Modern online analysis tools (Petoukhov *et al.*, 2007 ▶; Incardona *et al.*, 2009 ▶; Kieffer & Wright, 2013 ▶) provide a wealth of information (reduced one-dimensional curves, models *etc.*) on the measured samples. In order to proceed with the best course of action for each sample, it is important to combine these results for different measurements and to define criteria for sample quality, completeness and suggested further treatment. Here, the capability of ISPyB to compare results from different data-acquisition sessions and to combine them with information from different methods is clearly beneficial.

Additional data-acquisition modes in BioSAXS experiments can also be anticipated. One of them is online size-exclusion chromatography (SEC). SEC acquisition automatically provides a separation of mixtures, eliminates buffer mismatches and, as concentration varies during elution, comparison of individual time frames during the elution enables verification that the data are free of concentration-dependent effects. Although using standard HPLC columns typically represents lower throughput in terms of samples compared with standard acquisition, in some cases fast columns (*e.g.* Superdex 200 Increase 5/150 GL) can be used whose overall runtime is close to the duration of a complete standard acquisition dilution series. Data-acquisition parameters for SEC at BM29 are typically one frame per second (but five frames per second is achievable with the current setup) for the time the sample takes to elute (from 10 min to several hours depending on the type of SEC column and the flow rate). Online processing is required to provide real-time feedback in order to properly perform such experiments. Thus, screening all the acquired data and automated analysis results in a database that presents the relevant information and enables feedback on further experimental strategy.

## 2. Extension of ISPyB to BioSAXS   

### 2.1. Data model   

**Figure 1:**
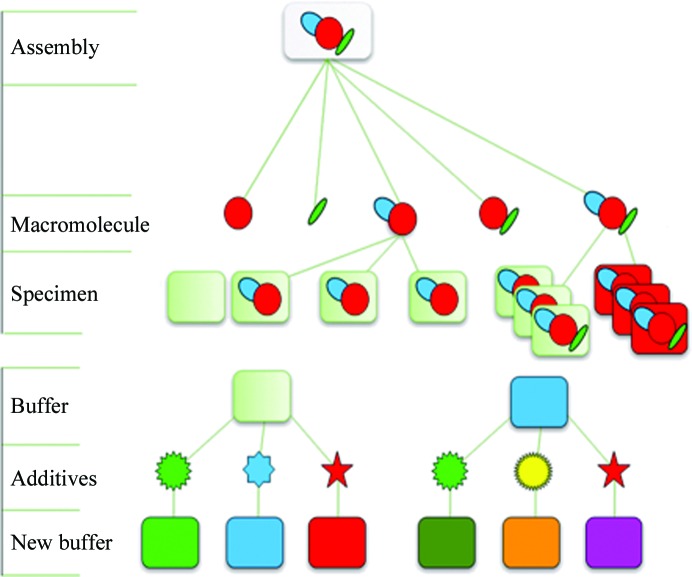
Sample hierarchy. Macromolecules (as a part of or as an entire assembly) can be measured as separate specimens in different buffer conditions or in the same buffer at different temperatures or time points.

**Figure 2:**
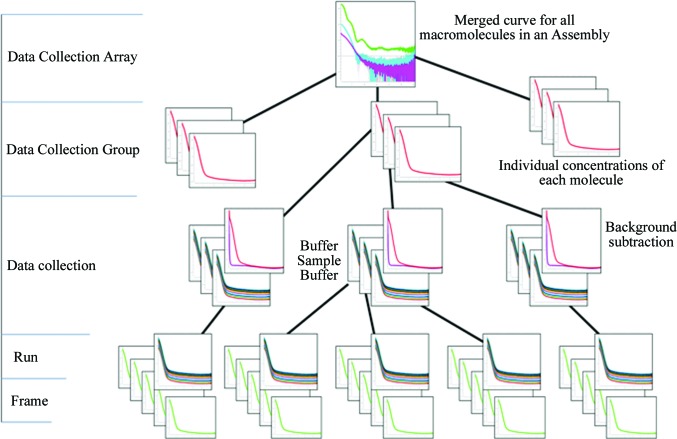
Data-collection hierarchy. Multiple frames are recorded per run and grouped into data collections with corresponding buffer measurements. Background subtractions of several individual macromolecule concentration measurements are part of a data collection group which can represent all macromolecules in an assembly within a data collection array. The idealized merged curve is based on various individual data collection groups.

The data model is a description of the tables and variables which will be populated and how they are stored and manipulated. An efficient and accurate data model is required to cover all of the variables which need to be stored without duplication. The key to this is hierarchical organization: see Fig. 1 ▶ for the hierarchy of samples and Fig. 2 ▶ for the hierarchy of data collection. The hierarchy described is mereological as it relates not only the individual parts to the whole (macromolecules in an assembly) but also multiple parts to each other (macromolecules may be a combination of both macromolecules and part of the assembly) and the whole. This system ensures that any variables used multiple times are stored at an appropriate level and are consequently linked to all instances where required. In order to achieve this goal, the experiment and its terminology have to be defined.

#### 2.1.1. List of terms: biological hierarchy  

*Macromolecule*: biological construct in solution for investigation.
*Buffer*: the solution in which a particular macromolecule is suspended.
*Specimen*: data-acquisition details for any volume of solution, including links to the buffer and/or the macromolecule, if present, plus its concentration.
*Additive*: any component of the buffer which will be varied in an experiment (salts, ligands, detergents lipids, deuteration).
*Assembly*: description (size, composition) of a complex containing multiple macromolecules (different combinations of individual macromolecules are considered as a separate macromolecules themselves; see the example in §[Sec sec2.1.2]2.1.2). The assembly definition includes the option to define the stoichiometry, as the binding of individual macromolecules in the assembly cannot always be assumed to be 1:1.
*Experiment*: list of related assemblies, macromolecules and their results which are used in different sessions.

#### 2.1.2. List of terms: data-classification hierarchy  

*Frame*: one individual exposure of a detector (refers to both the raw detector image and the reduced one-dimensional curve).
*Frameset*: all frames acquired simultaneously (corresponding to the same timepoint) by different detectors.
*Measurement/run*: all frames/framesets for an individual acquisition of a specimen.
*Data collection*: combination of runs for all specimens required to obtain the background-subtracted scattering of the macromolecule alone (blank-before, macromolecule at one concentration and blank-after).
*Data collection group*: combined data collections for one macromolecule (a minimum of three concentrations).
*Data collection array*: the data collection groups that are required to answer a biological question.
*Session*: a slot of beamtime allocated to a particular proposal (may contain multiple data collection arrays).

Depending on the complexity of the system, for some cases (for example structure validation) a data collection array will only contain one data collection group. However, for others there could be many groups in the array.

#### 2.1.3. Example of possible experiments   

**Figure 3:**
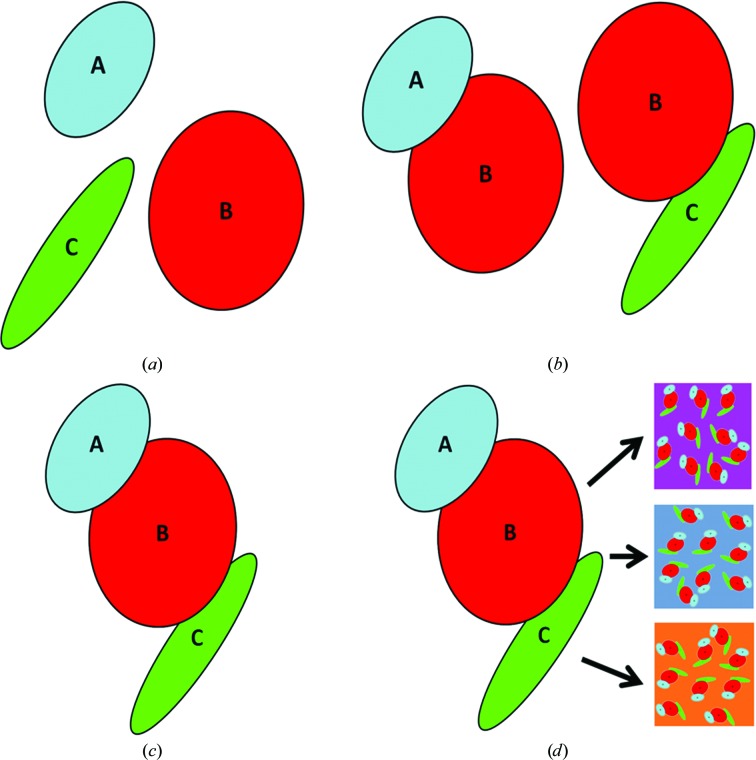
In order to determine the structure of the trimeric complex ABC, several samples need to be measured: (*a*) the monomers A, B and C, (*b*) the dimeric complexes AB and BC and (*c*) the trimeric complex (assembly) ABC; (*d*) the complex ABC is then measured in different buffer conditions containing the ligands/additives required for activity and/or nonhydrolysable substrate analogues to isolate the various stages of the reaction.

An example enzyme which in its functional form is comprised of three individual subunits A, B and C is schematically depicted in Fig. 3 ▶(*a*). The subunits can form a dimeric complex (Fig. 3 ▶ *b*) and a trimeric complex (Fig. 3 ▶ *c*). The first part of the experiment, P1, is to determine how the subunits fit together. Thus, the individual macromolecules A, B and C as well as the dimeric complexes AB and BC and finally the trimeric complex will be measured individually under the same buffer conditions. The second part of the experiment, P2, is to understand how the enzyme functions and will comprise data collections for the trimeric complex ABC (in the same conditions as for P1) plus additional data collections for different buffer conditions (Fig. 3 ▶ *d*).

The data model was designed with consideration of stoichiometry, as it cannot be assumed that there is only one binding site and therefore a 1:1 ratio between all macromolecules in the assembly. It is very common for there to be multiple binding sites and therefore 2:1, 3:1 or any other ratios can be present. The option for stoichiometry is also useful for macromolecules which may form different types of oligomers. Simple and accurate definition of the possible stoichiometry is therefore essential to facilitate the analysis; appropriate checks for mixtures and fitting of the model(s) may be automatically included in downstream analysis.

### 2.2. Integration with the MX data model   

**Figure 4:**
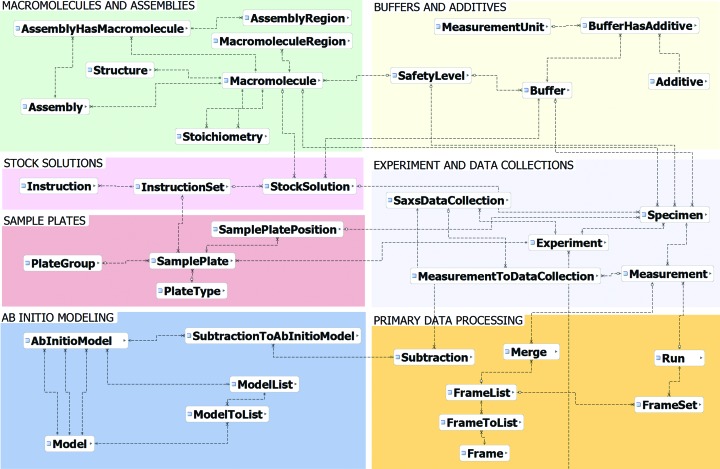
Schematic representation of the ISPyBB data model. The image has been divided into modules in order to show the tables in a logical layout. Different modules have been labelled in grey with different background colours for clarity. The data tables correspond to the labelled boxes in blue, with their relations shown by lines. Table fields are excluded for clarity.

As BioSAXS experiments necessitate different information to be stored in the database from MX experiments, the data model, its tables and their values have to be modified (Fig. 4 ▶). However, the overall layout of the extension is similar and as there are parts which are the same or similar, such as shipping, many tables have been reused.

Although BioSAXS is the first extension to be added to ISPyB, care was taken to ensure that the data model can be further extended. Additional experiment types can be included in order to anticipate future experiments undertaken at the partner beamlines.

## 3. ISPyBB graphical user interface   

### 3.1. Experiment preparation from the home laboratory   

**Figure 5:**
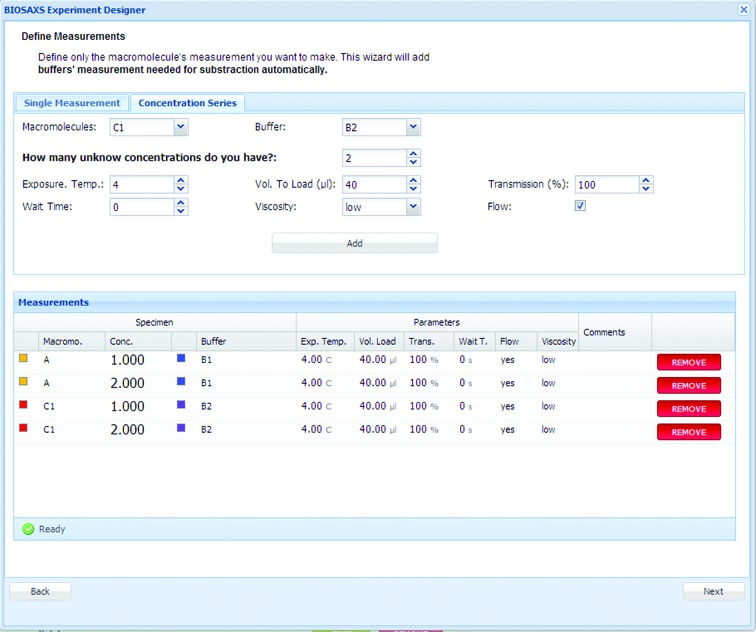
Prepare Experiment table: a template for single measurements or concentration series to fill.

Users are encouraged to prepare their experiments by providing information for the ‘Prepare Experiment’ table. The macromolecules and corresponding buffers are defined in single measurements or series of concentrations using a template (Fig. 5 ▶). Experimental parameters, such as quantity to be loaded, concentration, exposure temperature, position in sample changer *etc.* are also included. The list of measurements to be performed is created (Supplementary Fig. S4*a*), including the specimen list and their positions in the plate (Supplementary Fig. S4*b*). Based on this information, the required sample volumes are calculated (Supplememtary Fig. S4*c*), allowing users to prepare a sufficient quantity of samples or to modify their data-acquisition strategy well before the experiment.

### 3.2. Remote visualization of experimental status   

**Figure 6:**
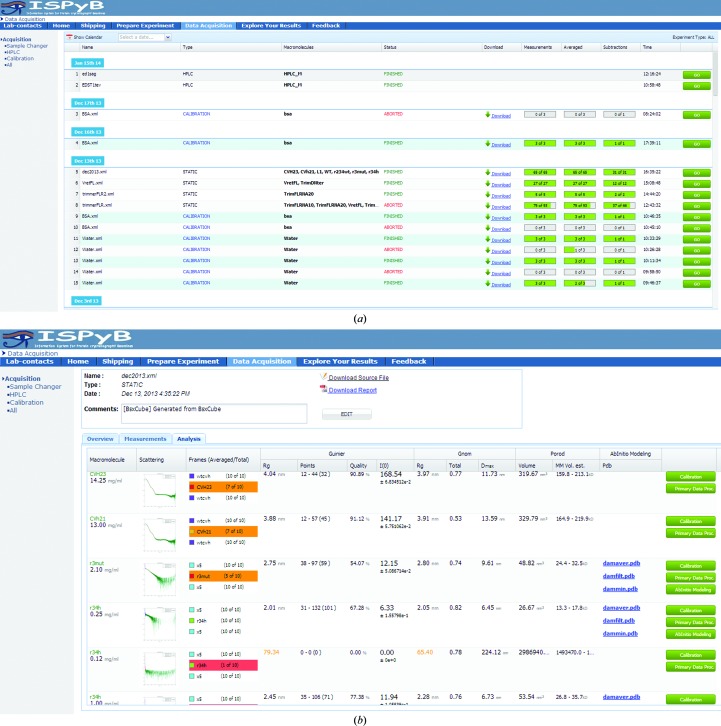
Data Acquisition table: (*a*) showing the summary of all experiments and their status and (*b*) the ‘Analysis’ window highlighting problems with data collections as variations between subsequent frames and discrepancy in *R*_g_ obtained either from Guinier or *GNOM* analysis using colour coding (orange, gentle warning; red, serious warning).

The status of a given experiment can be followed directly on the beamline or remotely by users at home. Once a user (or a beamline operator) has logged into the BCM (Beamline Control Module) connected to ISPyBB, data acquisitions are immediately recorded in the database, archived and displayed in the ‘Data Acquisition’ table (Fig. 6 ▶ *a*). Data acquisitions performed by the same user (*i.e.* with the same experimental logging) are divided into sessions according to the date when experiments were (or are) performed. There are currently two main types of experiments carried out at synchrotron-based BioSAXS facilities: data acquisition using a sample changer robot or a SEC system, which are denoted as STATIC or HPLC types in the Data Acquisition list, respectively.

When a sample-changer robot is used, a top-level summary displays the status (finished or aborted), the macromolecules involved and the percentage of measurements, averages and subtractions carried out. Once data acquisition and processing are complete, a ZIP file with the most relevant files of the data acquisition, including one-dimensional, average and subtracted curves, scattering, *P*(*r*), *GNOM* and Kratky plots, can be downloaded from the GUI. A lower level of information is accessible by clicking on a given data-acquisition line. For each sample, information is arranged within three different windows: Overview, Measurements and Analysis. The ‘Overview’ window (Supplementary Fig. S5) shows a list of specimens with their main parameters (macromolecule, buffer, concentration, volume in well and sample plate position) and an interactive image which describes schematically the arrangement of the specimen within the sample plates. The Measurement table gives further details of the measurements (exposure temperature, volume to load, transmission, flow, viscosity, energy and time per frame) and highlights completed measurements. The Analysis window (Fig. 6 ▶ *b*) displays a list of data collections, with remarks and warnings are found.

**Figure 7:**
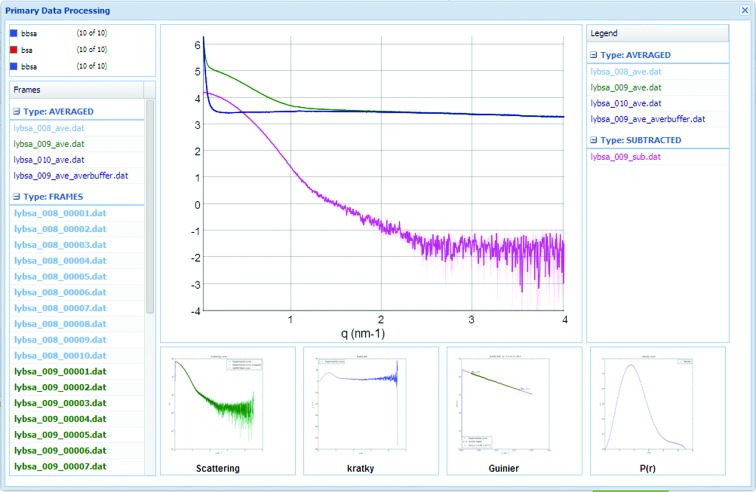
Primary data-processing window for a data collection (buffer–sample–buffer). Sample, buffer and subtracted curves are displayed together with Kratky, Guinier and *P*(*r*) plots.

Experimental results with primary data processing, including one-dimensional curves, average and subtraction, can be explored by clicking on the ‘Show primary data processing’ button of each data collection; a typical window is shown in Fig. 7 ▶. ISPyBB allows users to visualize, pan and zoom one-dimensional files, with no need for additional software. This feature is especially interesting when manual processing is required.

### 3.3. Integrated model visualization   

**Figure 8:**
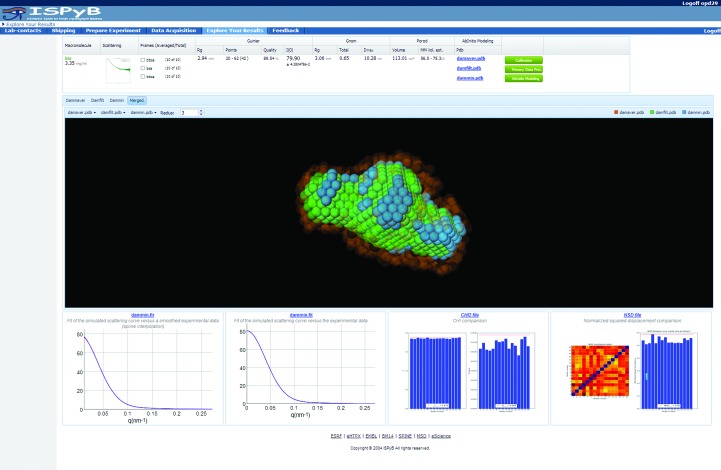
Ab Initio Modelling window showing the analysis results for the macromolecule and its *ab initio* reconstructed shape in an interactive three-dimensional rendering. Three models are shown: the average overlay (*DAMAVER*, orange), the most occupied volume (*DAMFILT*, green) and the *DAMMIN* final minimization (blue), which uses the average and filtered volume as a starting point. Close agreement between all these models indicates more robust reconstruction and thus minimal artefacts of flexibility/aggregation.

*Ab initio* models produced by online analysis are displayed in ISPyBB using a webGL visualization tool (http://www.khronos.org/webgl/) for *DAMMIN*, *DAMFILT* and *DAMAVER* models accessible in the ‘Analysis’ window of the Data Acquisition table after clicking on the ‘Ab Initio Modelling’ button (Fig. 8 ▶). To allow the estimation of the quality of a model, the fits of a simulated curve *versus* data, χ^2^ and nsd (normalized spatial discrepancy) plots are displayed. This initial visual inspection is for the purpose of verifying data quality. For publication, modelling should in general be performed manually and many individual modelling runs completed and averaged to produce viable and interpretable models for publication.

### 3.4. Additional experimental feedback   

Consistency in all measurements can be cross-checked, giving feedback on experimental artefacts such as radiation damage, buffer mismatches and cleanliness of the measurement cell. As the database contains the individual values, direct comparison between the molecules listed as being the same construct measured under similar conditions can be made.

### 3.5. Consistency in a single acquisition   

Solution data are assumed to be homogeneous, and in order to avoid radiation damage, samples are flowed through the beam during acquisition. However, if there is heterogeneity or radiation damage, the scattering observed during any measurements will vary. By comparing the number of similar frames with the total number acquired, it is clear whether the sample has variation which needs to be addressed, either by mitigating radiation damage or improving sample handling in the case of heterogeneity. To highlight problematic data, the corresponding frames are coloured orange as a warning if less than or equal to 70% are accepted and red if less than or equal to 40% of the frames are identical (Fig. 6 ▶ *b*). Additional data-analysis methods can be added to the values that are stored and displayed. The database is sufficiently flexible to allow future extensions and additional cross-checks can be easily added to further enhance the feedback to users.

### 3.6. HPLC   

**Figure 9:**
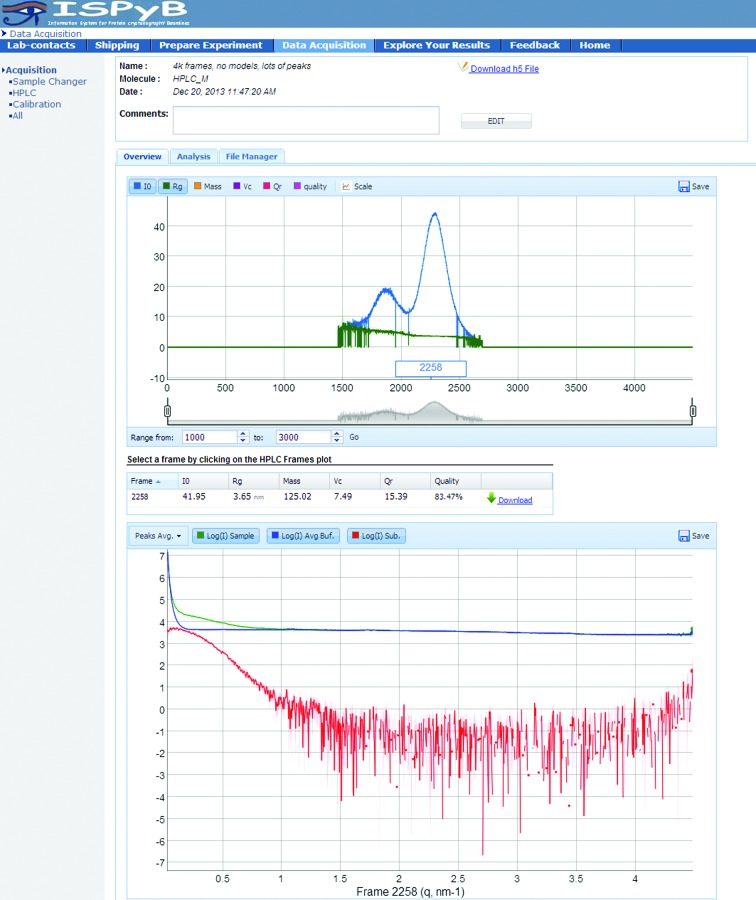
Overview of HPLC data in ISPyBB. Top, the interactive plot of *R*_g_ and *I*_0_ *versus* frame number to aid in the selection of frames to consider for further processing. Middle, the table of invariants for the selected frame. Bottom, the plot of the scattering data for the selected frame.

When an online SEC experiment is performed, ISPyBB provides an overview of the results of *AUTORG* (Petoukhov *et al.*, 2007 ▶), *I*_0_ and *R*_g_, as well as the mass estimate based on the approach of Rambo & Tainer (2013 ▶), for each individual frame. In this plot, users can easily identify regions of interest, *i.e.* experimental frames for further analysis (Fig. 9 ▶, upper graph). The lower graph of Fig. 9 ▶ (additional detail is given in Supplementary Fig. S6*a*) shows the SAXS curve of the buffer and the average signals of each peak found by the automatic processing pipeline. Users can also compare the experimental curves recorded at different times during sample elution by clicking on a given point of interest on the chromatogram. When selecting a point of interest, the table between the graphs displays the analysis results for this particular point.

In an analogy to experiments performed using a sample changer, the Analysis window of SEC measurements provides a list of the primary data-processing results of all merged data files, *i.e.* buffer and peaks, and access to the visualization of *ab initio* models for each peak.

Finally, the File Manager provides an easy-to-use interface to download all data files contributing to a merged data file or all data files in a region of interest (Supplementary Fig. S6*b*).

### 3.7. Concentration effects   

Variation between all corresponding samples (the same macromolecule under the same conditions) may be crosschecked by selecting the ‘Explore Your Results’ table (Supplementary Fig. S7*a*). ‘Good quality measurements’ (*i.e.* the data set is complete) are highlighted in green, ‘Probably valid with manual processing’ in orange and ‘More measurement needed to be done’ in red if further dilutions in the concentration series are needed. All measurements performed on a given macromolecule can be accessed by clicking the green ‘GO’ button on the right. A comparison of all measured concentrations is displayed in the Concentration Effects window (Supplementary Fig. S7*b*).

The ‘Explore Your Results’ tab can be also used to highlight not only variations in the samples between measurements but different measurements of the same parameter from the same data to add to information on data quality and the robustness of calculations and confidence in the values given. An example is the comparison of molecular mass (MM) obtained from the measured *I*_0_ and from Porod volume estimates, which relates to the accuracy of concentration normalization. Another cross-check is between the radius of gyration, *R*_g_, as calculated from the Guinier approximation and from the inverse Fourier transform (*GNOM*), which relates to the reliability of the resulting *P*(*r*) function and any subsequent modelling. If a significant difference between these two values is detected they are highlighted in orange (Fig. 6 ▶ *b*).

### 3.8. ISPyB as an experiment file manager   

ISPyB is not a backup system and does not contain the raw scattering images collected in the experiment. However, as it does hold the one-dimensional processed curves it allows users to browse through these frames using a simple interface and to download the data for a particular project for manual reprocessing. Download is possible for an individual data collection and/or all data for a particular macromolecule from the ‘Explore Your Results’ section. This facilitates the archiving and sharing of experimental data between collaborators at multiple sites.

### 3.9. ISPyB as an integrated system   

As ISPyB for BioSAXS is an extension to the existing ISPyB for MX, it inherently contains the possibility of combining information from both techniques. Macromolecules used for MX and SAXS may be the same construct and if so will have the same acronym in the database. This facilitates increased automation of advanced analysis techniques, such as verification of crystal structures under physiological conditions or even rigid-body modelling of complexes when data are available from both techniques. Additionally, it is foreseen to enable users to search the database and provide all relevant data sets (both MX and SAXS), but visualization of this is not currently available in the GUI.

#### 3.9.1. Chain of custody   

ISPyB integrates all steps of a SAXS experiment from sample preparation to acquisition and analysis in a single database. This unified approach facilitates a complete ‘chain of custody’ in a single program from the original target to the final structure with cross-validation under physiological conditions.

## 4. Discussion   

ISPyBB is greatly appreciated by users and feedback is very positive, stating that ISPyBB provides users with greater independence during experiments (and between them) and provides confidence that all data required for further analysis have been collected within the limits of sample availability. The user feedback has also led to interest from other non­partner synchrotron-radiation facilities such as SOLEIL and MAX-lab to use ISPyB with specific requests for the BioSAXS extension. The efficiency of BioSAXS experiments is increasing thanks to the ability to prepare experiments in advance and the enhanced access to results from automatic analysis. User groups are more confident, relaxed and efficient during their experiments. ISPyBB will soon be installed and operational at partner sites, giving users the benefit of all its features for experiments at ESRF beamline BM29, PETRA III beamline P12 and Diamond beamline B21.

## 5. Related literature   

The following reference is cited in the Supporting Information for this article: Ginn *et al.* (2014 ▶).

## Supplementary Material

Supporting Information.. DOI: 10.1107/S1399004714019609/ba5219sup1.pdf
