# Supplementary material for: ISPyB for BioSAXS, the gateway to user autonomy in solution scattering experiments
Source: Acta Crystallogr D Biol Crystallogr. 2015 Jan 1;71(Pt 1):76–85. doi: 10.1107/S1399004714019609 (PMC4304688; doi:10.1107/S1399004714019609)
Supplement: Supplementary file 1 [file d-71-00076-sup1.pdf]

BIOLOGICAL CRYSTALLOGRAPHY

Volume 71 (2015)

Supporting information for article:

ISPyB for BioSAXS, the gateway to user autonomy in solution scattering experiments

Alejandro De Maria Antolinos, Petra Pernot, Martha E. Brennich, Jérôme Kieffer, Matthew W. Bowler, Solange Delageniere, Staffan Ohlsson, Stephanie Malbet Monaco, Alun Ashton, Daniel Franke, Dmitri Svergun, Sean McSweeney, Elspeth Gordon and Adam Round

S1. Mobile access

The ISPYBB web interface provides easy access to the information in the database from different places and different platforms. Recently, the incorporation of HTML5 technology allows ISPYBB pages to be accessed using mobile technology (both iOS and android) – allowing greater convenience for an increasingly connected user community. Users can prepare experiments; monitor their sample shipment; or view summary pages of the data collections from any device of their choice from any location with an internet connection (Ginn et al., 2013).

S2. Ancillary tools for Beamline Staff

In order to take full advantage of the logged experiments beamline staff tools have been provided to aid diagnostics and ensure reliability.

S2.1. Data mining

Data logging increases efficiency at all stages of an experiment (preparation, acquisition and analysis) by presenting the important information in an intuitive way. Integrating ISPyB for BioSAXS gives additional indirect benefits to the users. With the 'Data mining' tool beamline staff are informed of how the user community is using the beamline and how the beamline is performing. This allows feedback to ensure reliability and data quality, rapid response in case of problems and improved anticipation of the real needs of the community for future developments. Some essential feedback for beamline managers is shown in Figure S2.

S2.2. Logger

The ISPyB logger is a tool for developers, system administrators and beamline staff. It allows them to diagnose a problem even before users are aware of its existence. A typical example is shown in Figure S3, the view which highlights logins; communication between programs, which has highlighted a computer switched off and facilitated an immediate restart preventing data loss; errors, which has enabled the development team to be deploying the bug fixes when the local contact called to highlight an issue. The view also highlights time consuming methods among other statistics, allowing the developers to focus on issues directly affecting experiments. Any time saved diagnosing problems is of direct benefit to the users and monitoring of how the systems is used under real conditions enables optimisation giving reliability and proactively preventing lost time.

S3. Technical specifications

ISPyBB is written in Java J2EE and uses the JBoss Application Server (http://jboss.org/jbossas). It combines Struts Model-View-Controller (MVC) Web Application Framework (http://struts.apache.org) and pure JavaScript using the library Ext JS (www.sencha.com) which corresponds to HTML5 standards. The end-user interface is a pure web-based application that is

accessible from any compatible browser. At the ESRF the database used for ISPyBB is MySQL (http://www.mysql.com) which is an open-source relational database and distributed under the terms of the GNU General Public License. At the DLS the database used is Oracle (http://www.oracle.com). ISPyBB uses ejb3s with Hibernate and/or ejbql as an object-relational mapping tools for the Java language, providing a framework for mapping an object-oriented model to a relational database.
In BioSAXS experiments the data visualization is crucial for interpreting the results. For ISPyBB a set of visualizations and plots has been developed. Once results have been analyzed by the online data analysis pipeline EDNA (Incardona et al, 2009), the ISPyBB database is populated through the web services and the user interface makes such results accessible in real time. For data visualization it has extended the use of Java Applets or other technologies like Flash or Silverlight where the installation of third-party plugging of software is necessary. However, in ISPyBB all data representation is carried out by the browser avoiding the need of installing external software taking the advantage of using HTML5 standard including SVG (Scalar Vector Graphics) and WebGL (Web Graphics Library). A current limitation is that HTML5 based applications can be run only in computers with recent graphics cards and with compatible web browsers.

S3.1. Sustainability

ISPyB is anticipated to be in long term use at multiple sites and therefore maintenance and support of the technologies used is of issue. The GUI has been developed using an inheritance-based design in order to move from one library to another in the case it becomes necessary in the future to move away from extJS. The database structure is in principle not restricted and already is adapted for use with Oracle and Mysql databases. Oracle has committed that MySQL will remain open source until 2015. However, there is no guarantee after this. For the facilities using a MySQL DataBase, the adaptation to Oracle already exists and switching should therefore be less difficult. Additionally a good choice could be MariaDB (https://mariadb.com/kb/en/mariadb-versus-mysql-compatibility/) which is a drop-in replacement to MySQL. When matching the versions of MySQL and MariaDB we would simply need to uninstall MySQL and install MariaDB which should require minimal effort.

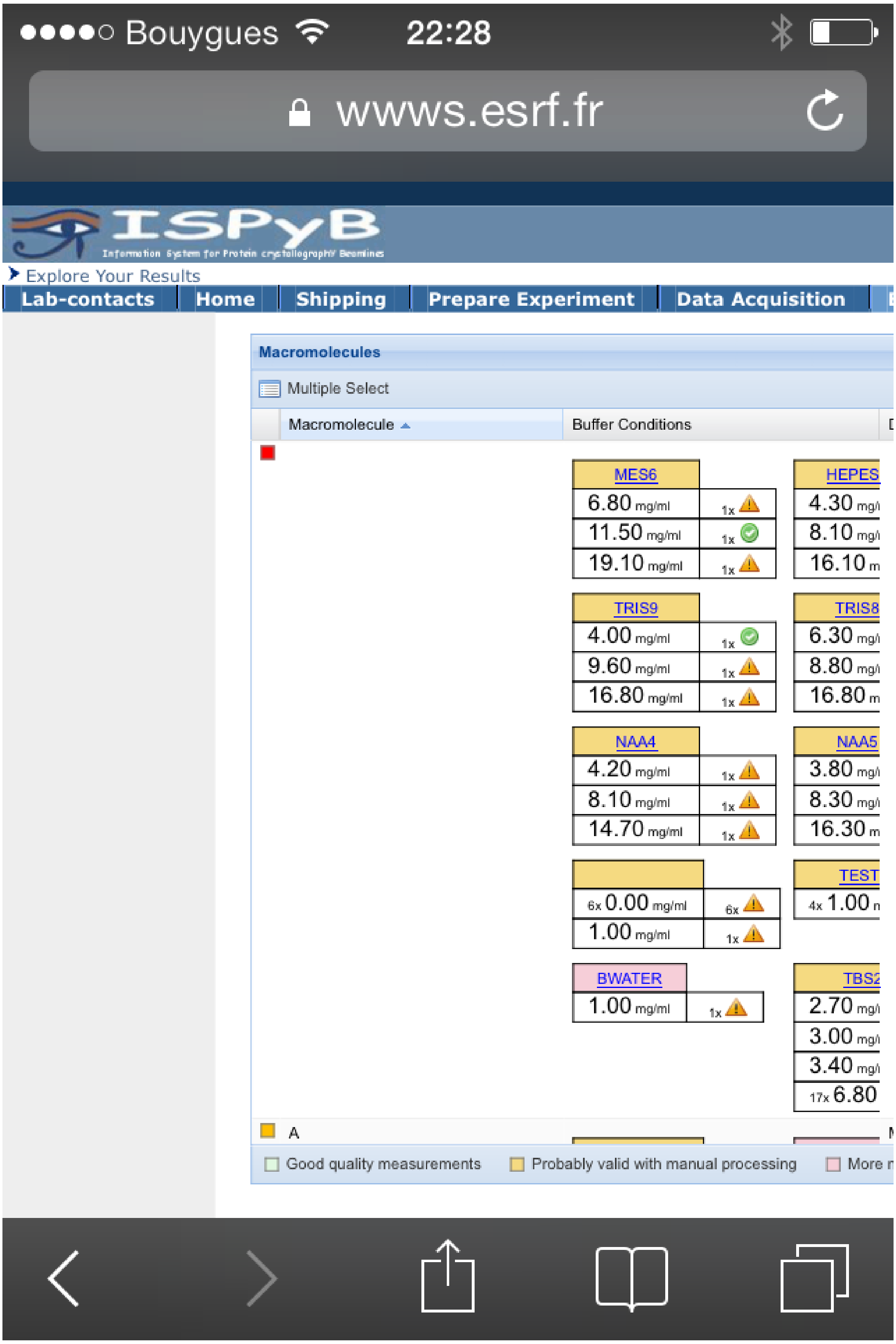

Bouygues
22:28
wwws.esrf.fr
ISPyB
Information System for Protein crystallography Beamlines
Explore Your Results
Lab-contacts
Home
Shipping
Prepare Experiment
Data Acquisition
Macromolecules
Multiple Select
Macromolecule
Buffer Conditions
MES6
6.80 mg/ml 1x
11.50 mg/ml 1x
19.10 mg/ml 1x
HEPES
4.30
8.10
16.10
TRIS9
4.00 mg/ml 1x
9.60 mg/ml 1x
16.80 mg/ml 1x
TRIS8
6.30
8.80
16.80
NAA4
4.20 mg/ml 1x
8.10 mg/ml 1x
14.70 mg/ml 1x
NAA5
3.80
8.30
16.30
6x 0.00 mg/ml 6x
1.00 mg/ml 1x
TEST
4x 1.00
BWATER
1.00 mg/ml 1x
TBS2
2.70
3.00
3.40
17x 6.80
A
Good quality measurements
Probably valid with manual processing

(a)

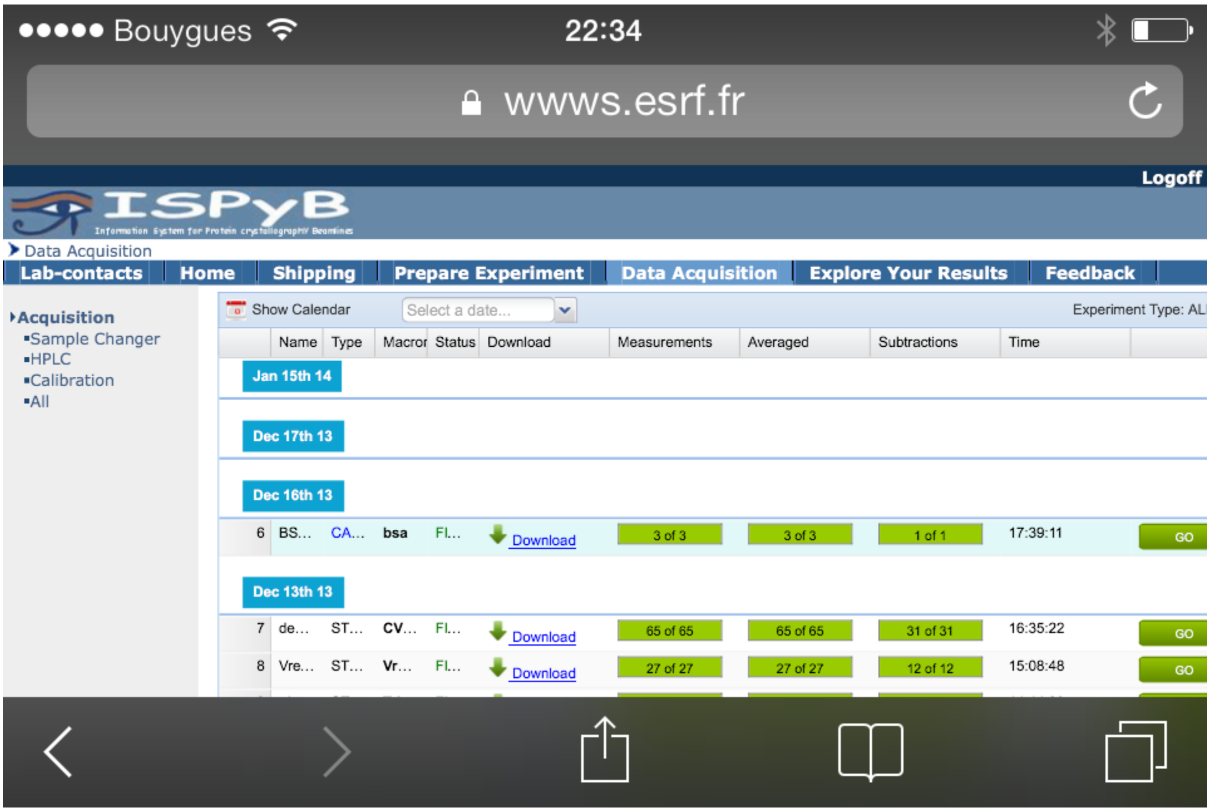

(b)

Figure S1 Mobile view using Safari on iPhone a) Explore results, b) Data collection.

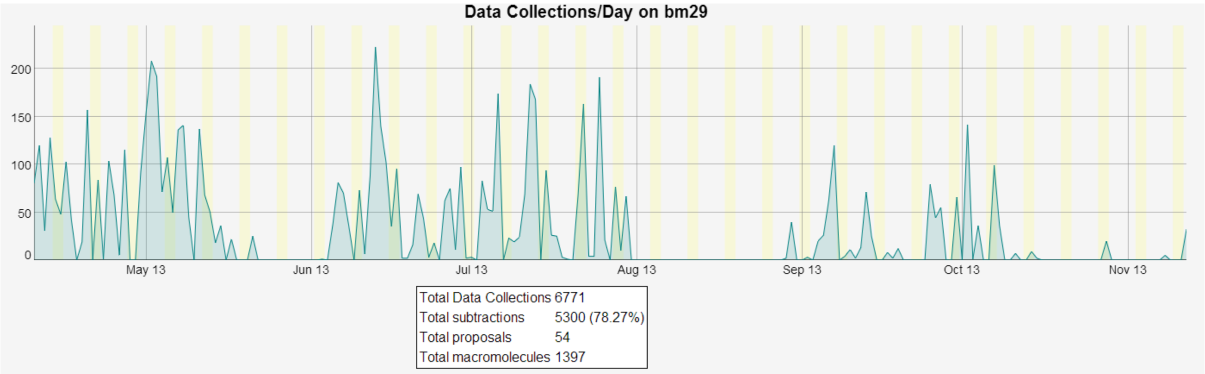

Figure S2 Examples of 'Data mining' table for beamline managers, Data Collections per day table displays productivity of the beamline.

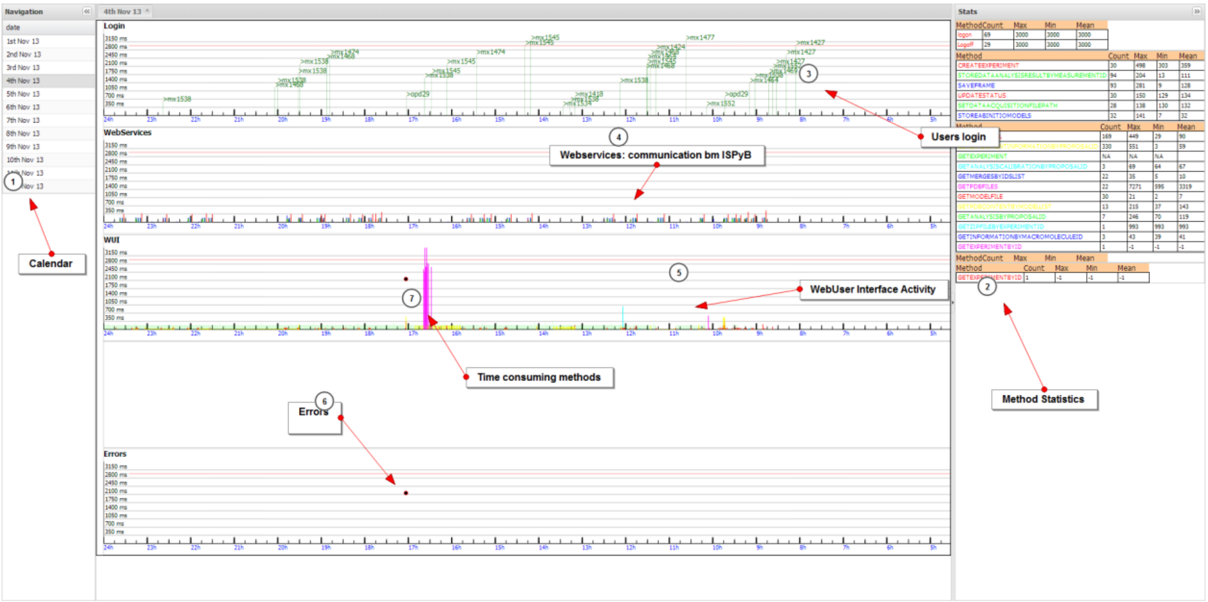

Figure S3 Example for the use of the logger tool for monitoring our webservices. 1) Calendar to select dates to view, 2) statistics of methods used, 3) User login times, 4) times for Beamline ISPyB communications 5) User activate on Web interface, 6) Errors (highlighting problems which needs to be fixed), 7) time consuming methods (in need of further optimisation).

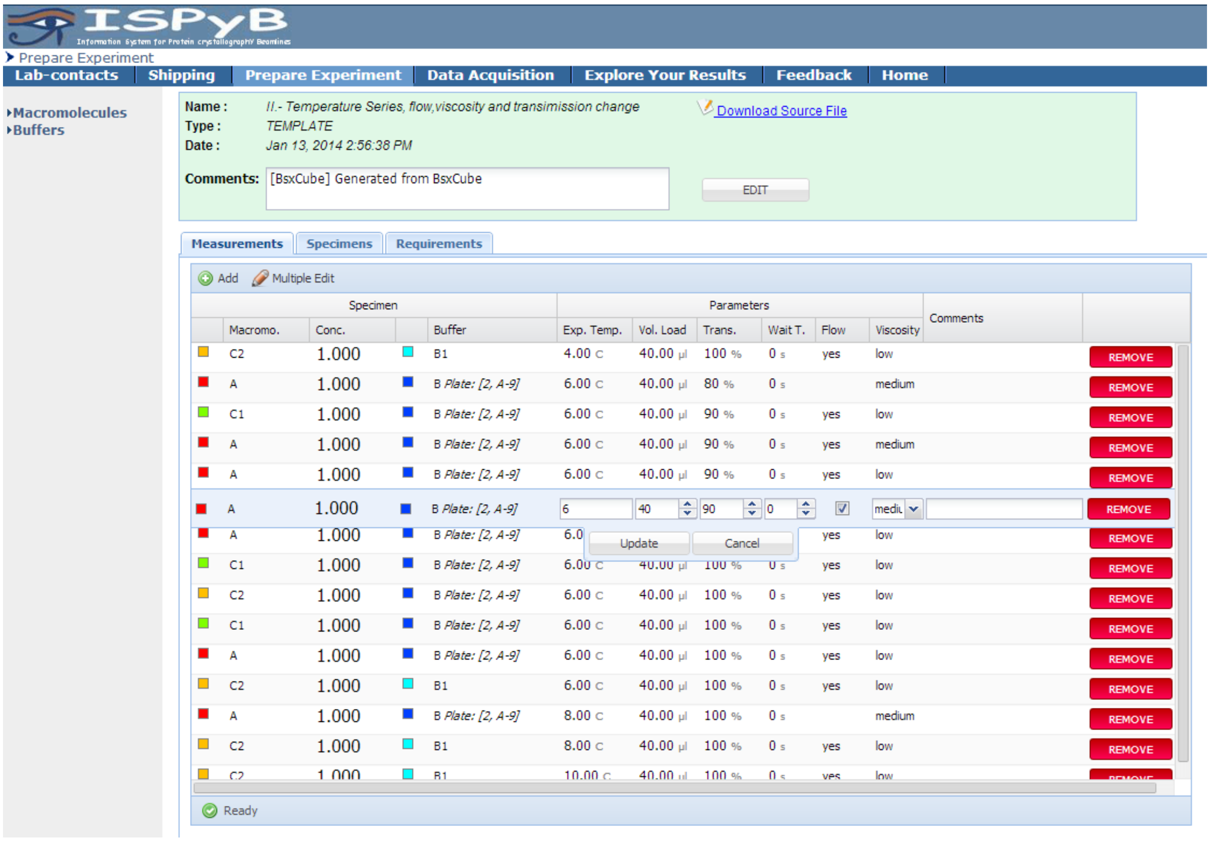

(a)

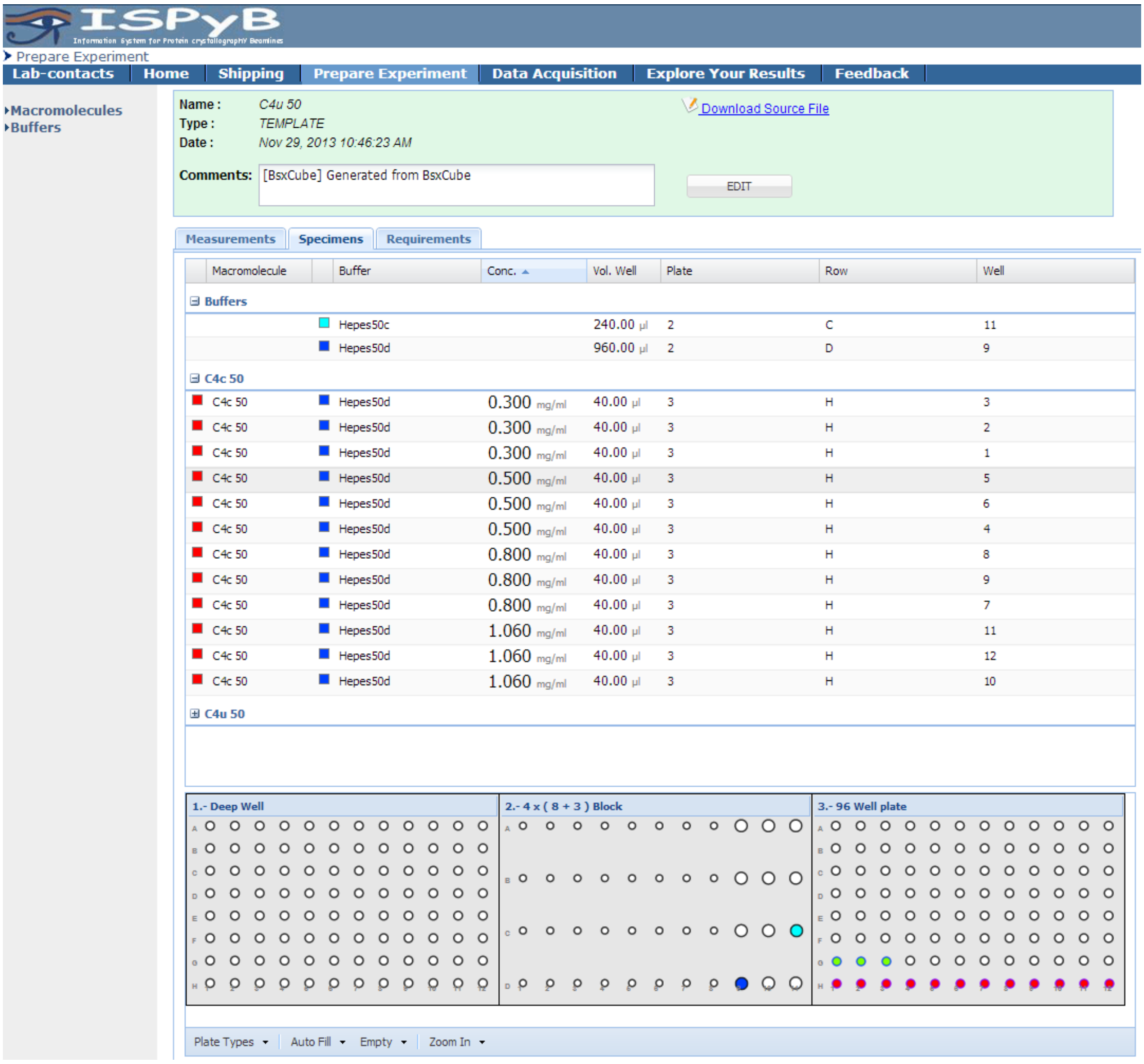
ISPyB
Information System for Protein crystallography Beamlines
Prepare Experiment
Lab-contacts
Home
Shipping
Prepare Experiment
Data Acquisition
Explore Your Results
Feedback
Macromolecules
Buffers
Name : C4u 50
Type : TEMPLATE
Date : Nov 29, 2013 10:46:23 AM
Download Source File
Comments: [BsxCube] Generated from BsxCube
EDIT
Measurements
Specimens
Requirements
Macromolecule
Buffer
Conc.
Vol. Well
Plate
Row
Well
Buffers
Hepes50c 240.00 µl 2 C 11
Hepes50d 960.00 µl 2 D 9
C4c 50
C4c 50 Hepes50d 0.300 mg/ml 40.00 µl 3 H 3
C4c 50 Hepes50d 0.300 mg/ml 40.00 µl 3 H 2
C4c 50 Hepes50d 0.300 mg/ml 40.00 µl 3 H 1
C4c 50 Hepes50d 0.500 mg/ml 40.00 µl 3 H 5
C4c 50 Hepes50d 0.500 mg/ml 40.00 µl 3 H 6
C4c 50 Hepes50d 0.500 mg/ml 40.00 µl 3 H 4
C4c 50 Hepes50d 0.800 mg/ml 40.00 µl 3 H 8
C4c 50 Hepes50d 0.800 mg/ml 40.00 µl 3 H 9
C4c 50 Hepes50d 0.800 mg/ml 40.00 µl 3 H 7
C4c 50 Hepes50d 1.060 mg/ml 40.00 µl 3 H 11
C4c 50 Hepes50d 1.060 mg/ml 40.00 µl 3 H 12
C4c 50 Hepes50d 1.060 mg/ml 40.00 µl 3 H 10
C4u 50
1.- Deep Well
2.- 4 x (8 + 3) Block
3.- 96 Well plate
Plate Types
Auto Fill
Empty
Zoom In

(b)

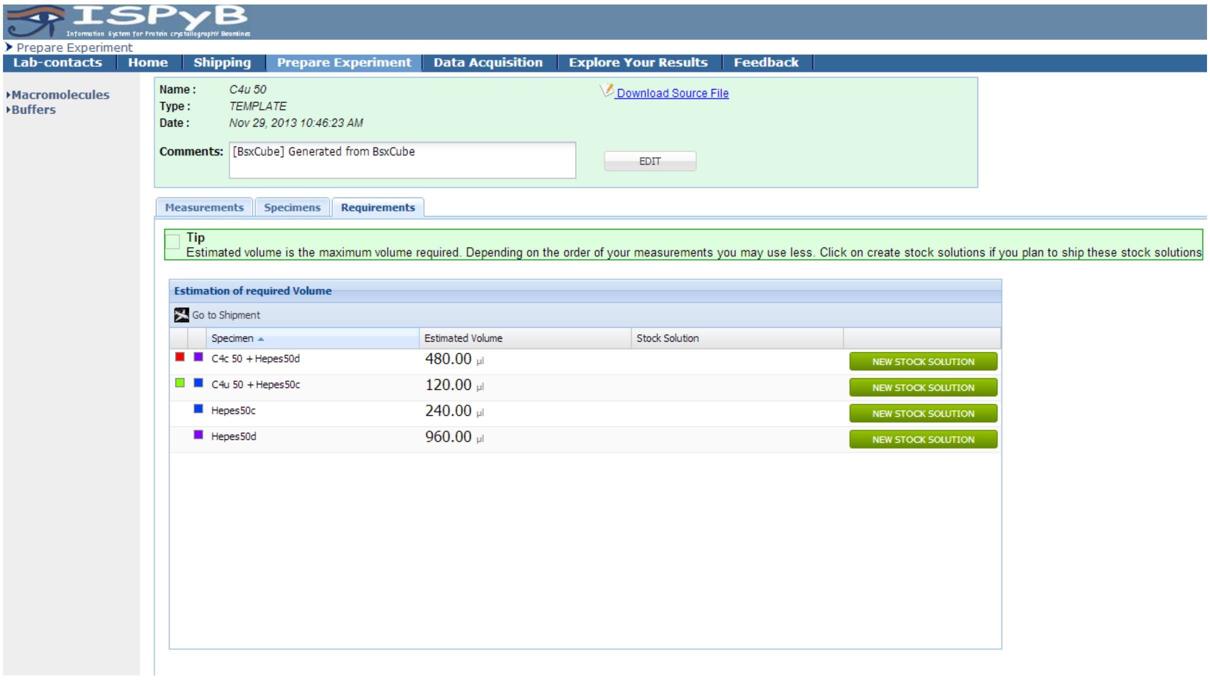

(c)

Figure S4 *Prepare Experiment* table: a) list of measurements created, b) specimen list with its locations in sample changer, c) sample requirements window.

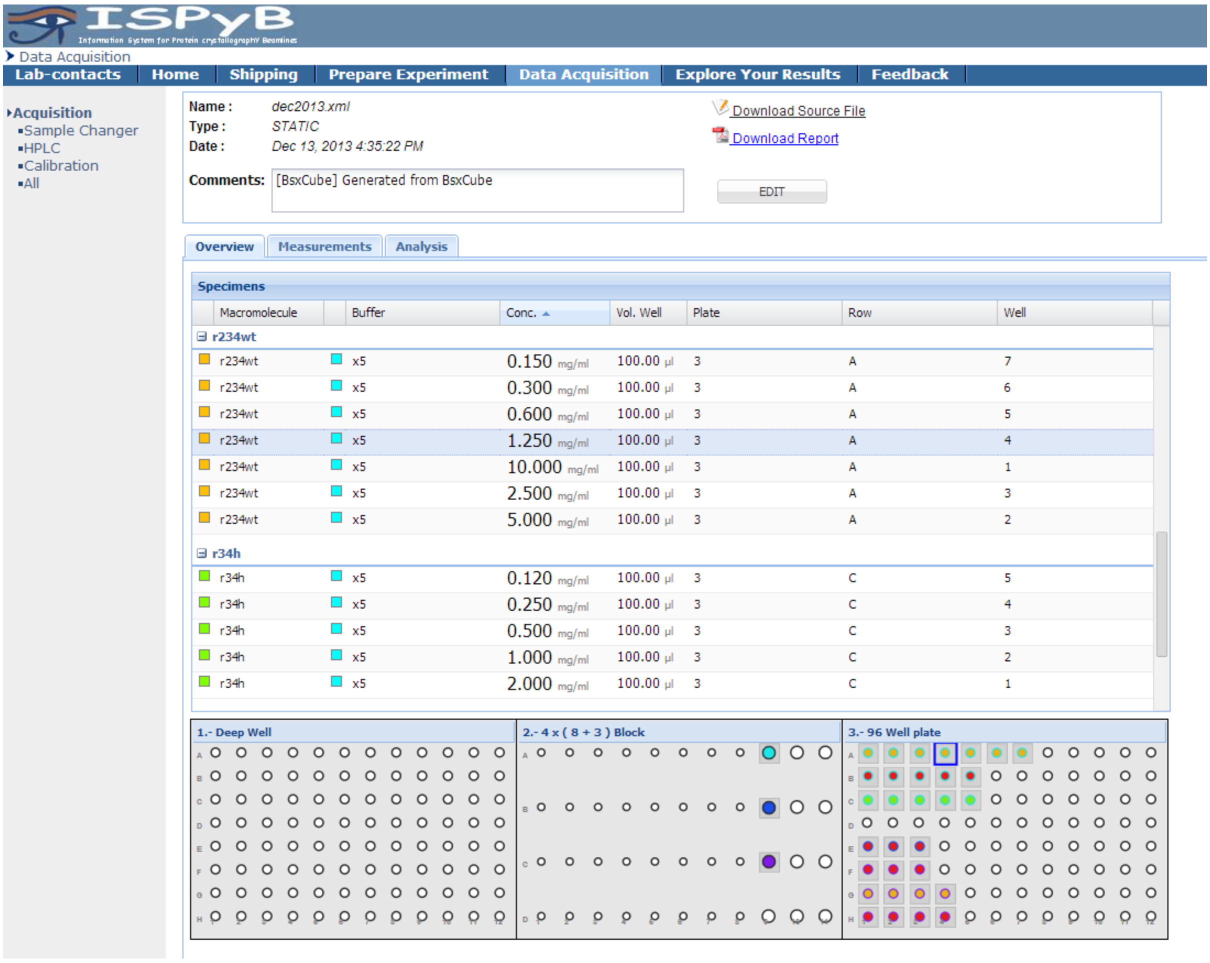

Figure S5 Summary of each experiment showing the samples to be measured and their locations in the sample changer.

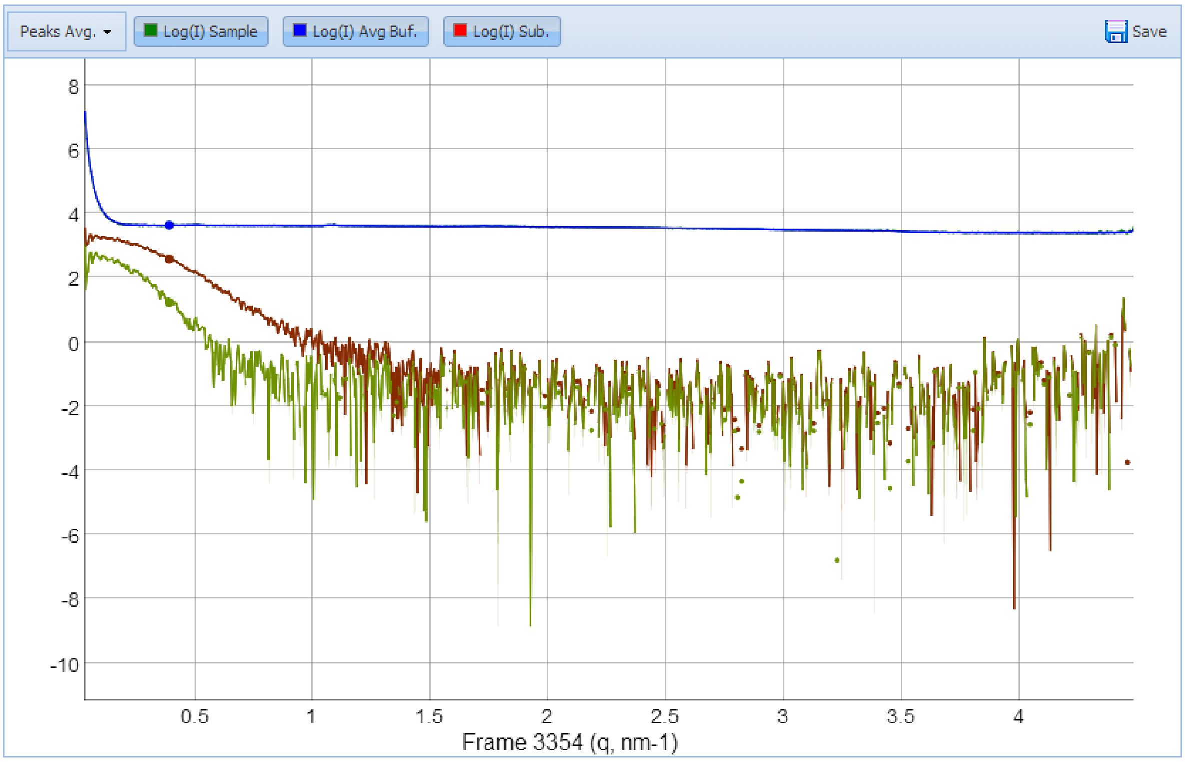

(a)

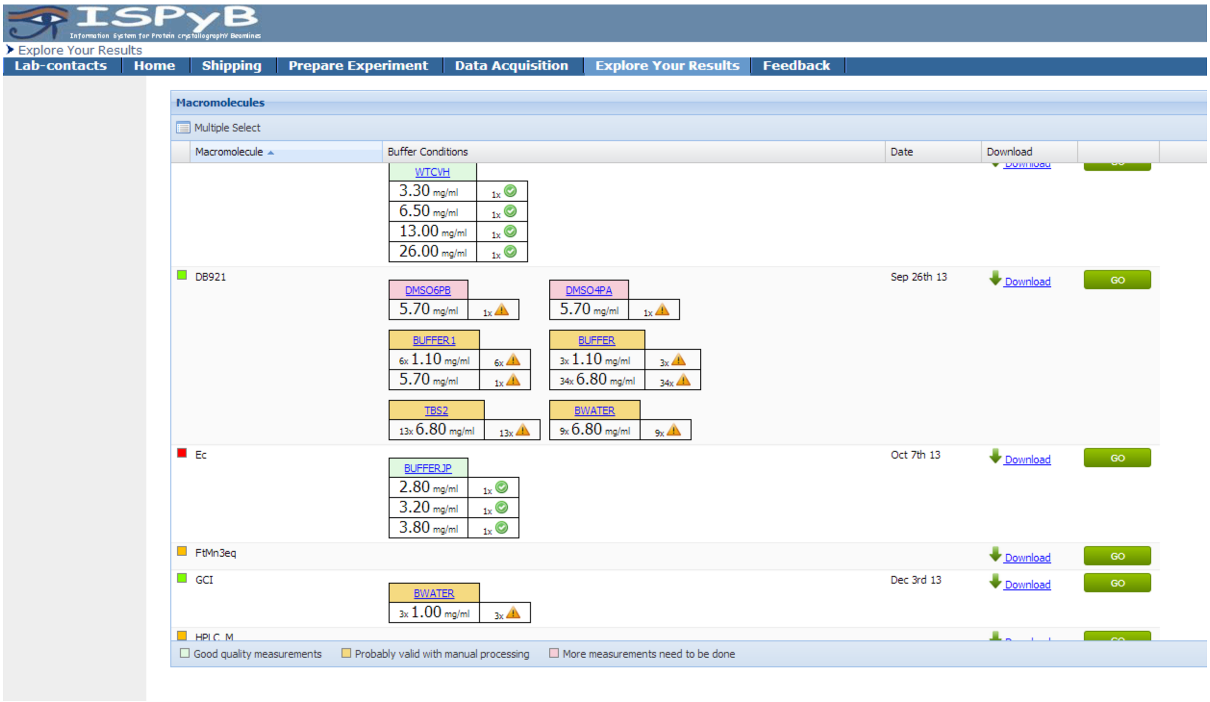

(b)

Figure S6 a) 1D plot of the scattering data for the merged files produced by the auto processing, blue averaged buffer used for subtractions, Brown and green show the merged profiles for the peaks detected. b) File manager, shows the peaks detected and the frames used as well as a download option for each peak.

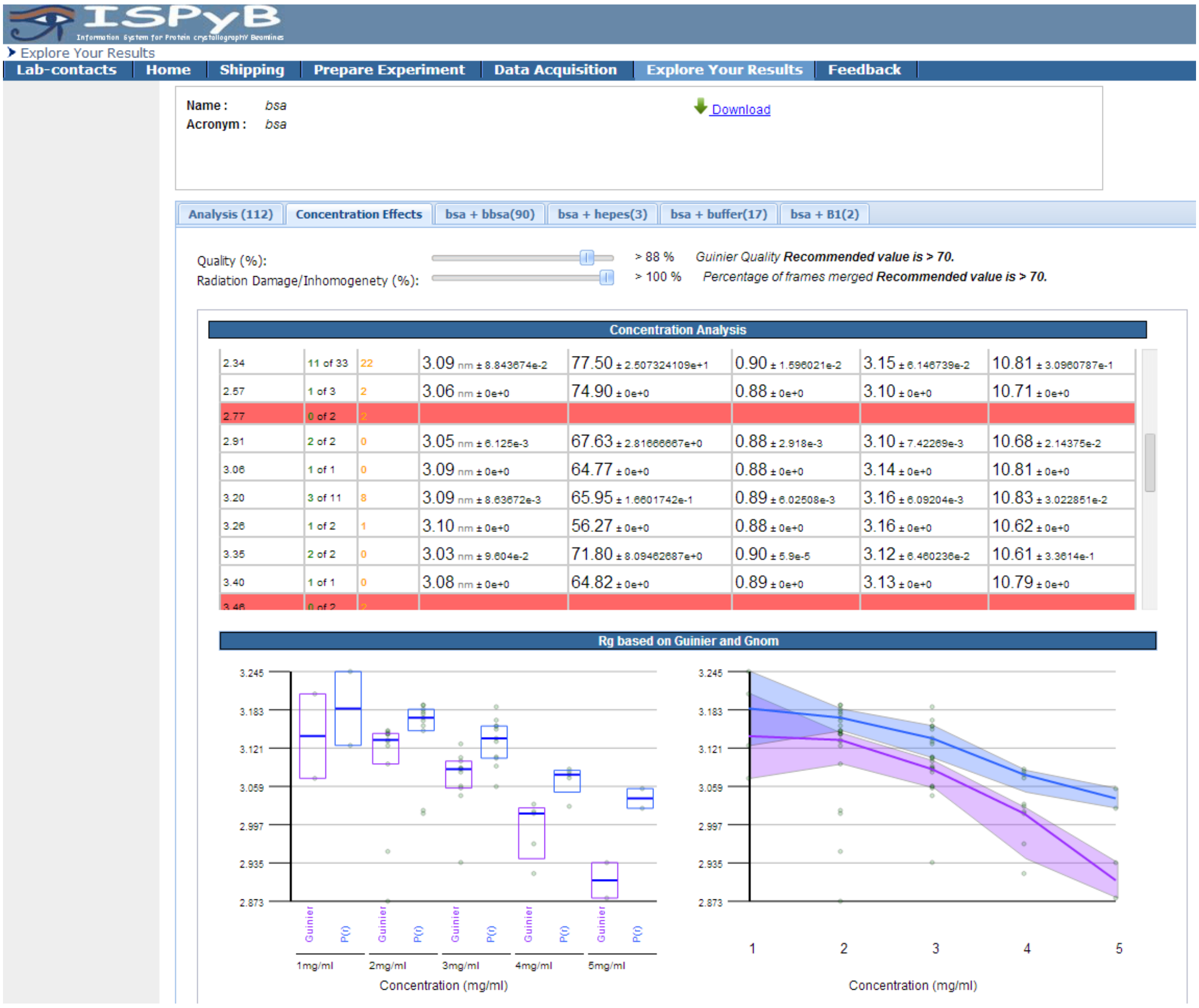

Figure S7 *Explore Your Results table*: a) general view of all measured compounds, b) Concentration Effects window for a given macromolecule.

Supplementary References

Ginn, H., Mostefaoui, G.K., Levik, K., Grimes, J.M., Walsh, M.A., Ashton, A.& Stuart, D.I. (2013) *CCP4 Newsletter on protein crystallography*, **49**, 20-24
